# Successful use of cannabidiol in nonconvulsive status epilepticus in Angelman syndrome

**DOI:** 10.1002/epi4.12948

**Published:** 2024-08-22

**Authors:** Nicola Pietrafusa, Luca De Palma, Michelina Armando, Tiziana Corsetti, Federico Vigevano, Nicola Specchio

**Affiliations:** ^1^ Neurology, Epilepsy and Movement Disorders Unit, Full Member of European Reference Network EpiCARE Bambino Gesù Children's Hospital, IRCCS Rome Italy; ^2^ Department of Neurorehabilitation Bambino Gesù Children's Hospital, IRCCS Rome Italy; ^3^ Hospital Pharmacy Unit Bambino Gesù Children's Hospital, IRCCS Rome Italy; ^4^ Neurological Sciences and Rehabilitation Medicine Scientific Area Bambino Gesù Children's Hospital, IRCCS Rome Italy

To the Editors

Angelman syndrome (AS) is a rare neurogenetic disorder characterized by developmental delay, epileptic seizures, cognitive impairment, electroencephalographic epileptiform and slow interictal abnormalities, and motor dysfunction.^1^

In AS, nonconvulsive status epilepticus (NCSE) is frequent, is characterized by period of decreased responsiveness which may last hours to days, and it occur in about 20% of patients.^2^ Treatment of NCSE in AS is challenging and no specific drugs are approved with this purpose.

Epidyolex® is approved by EMA up to a dose of 20 mg/kg/d for individuals >2 years with Lennox–Gastaut Syndrome (LGS) or Dravet Syndrome (DS), and with a higher maximum dose of 25 mg/kg/d in those with tuberous sclerosis complex (TSC) (EMA).^3^

We recently treated an 8‐year‐old boy with AS expressing deletion of 15q11.2q13 (6.23 Mb). At the age of 2 years, he started to present with asynchronous bilateral upper limbs myoclonia. He was treated with clonazepam and ethosuximide with good effects, being almost seizure‐free until the age of 5 years, when myoclonia associated with poor responsiveness reappeared consistently.

At the age of 8 years, he was receiving ethosuximide (20.5 mg/kg/d) and clonazepam (0.08 mg/kg/d), he presented with marked drowsiness and an increase of myoclonia (Figure 1A). He was admitted in our Department of Neurology (Bambino Gesù Children Hospital ‐ Rome, Italy). Long‐term EEG monitoring showed a NCSE pattern (Figure 1A,B), clinically characterized by a reduction in motor initiative and an increase in tremor. This pattern resolved only intermittently during intravenous Midazolam administration. Intravenous valproate (bolus at 30 mg/kg/d and then continuous infusion at 2 mg/kg/d) (Figure 1C) and levetiracetam (bolus at 60 mg/kg/d) (Figure 1D), were ineffective and therefore stopped.

**FIGURE 1:**
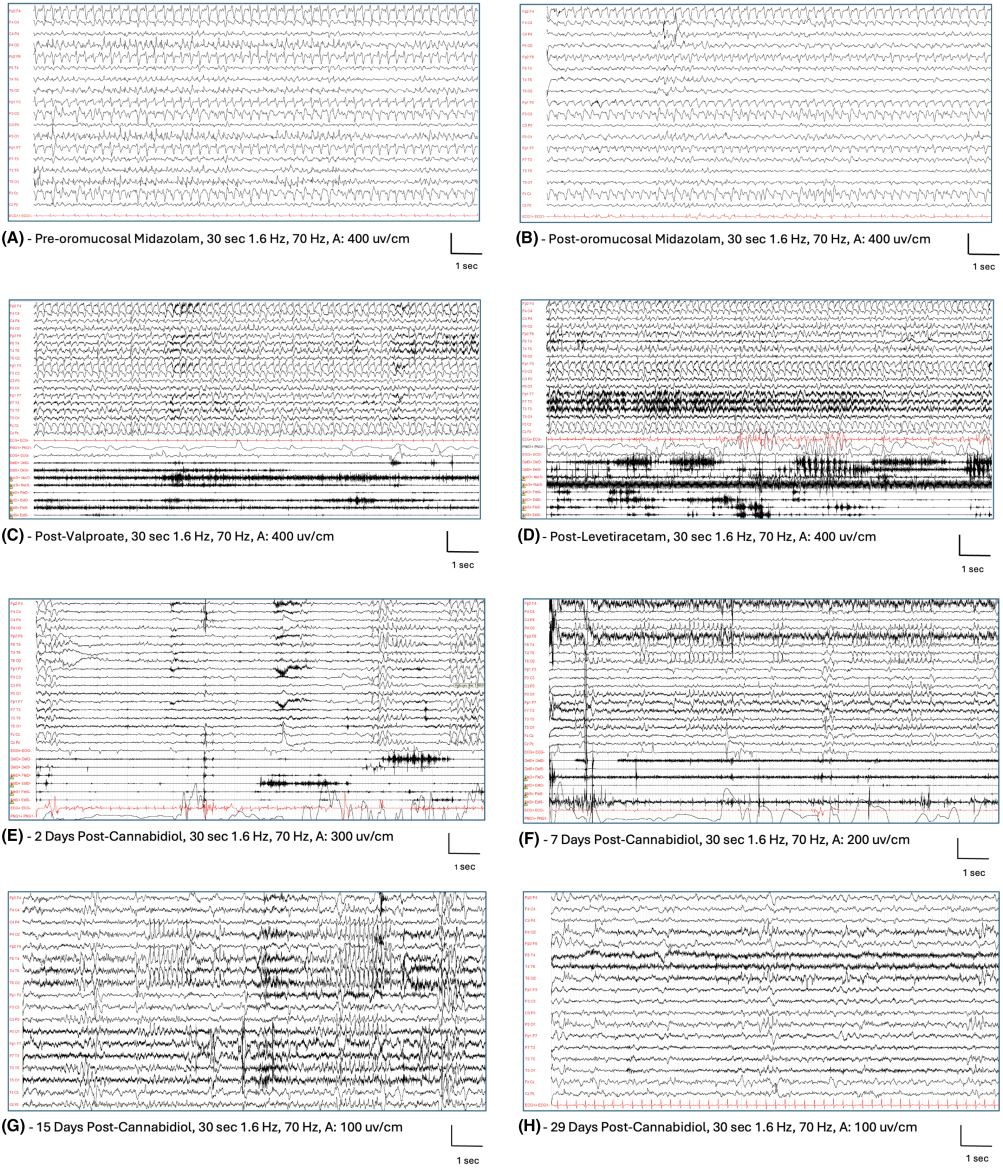
EEG during the drowsiness and frequent seizures. This patient was recorded few days after the symptoms began. Continuous high amplitude, diffuse, irregular spike–wave complexes with higher amplitude over bilateral frontal regions, in sequences with a rhythmic‐recruiting pattern (A). Oromucosal midazolam was administered without significant changes (B). After 1 day EEG still showed continuous spike and waves discharges, EMG channels show multiple asynchronous and asymmetric myoclonic jerks over bilateral deltoids, and neck muscles. The patient was also presenting head drops typically seen in NCSE (minor motor status). Both valproate (C) and levetiracetam (D) were ineffective. He started Epydiolex CBD and after 2 days (E) and 7 days (F) there was a marked reduction of EEG epileptiform abnormalities and a resolution of NCSE. After 15 days (G) still some repetitive spikes were evident over asynchronous bilateral temporal and parietal regions. After 29 days (H), there was an improvement of background activity, further reduction in epileptiform abnormalities and the patient was persistently free from seizures.

We added Epidyolex® CBD, with a faster titration than usual, starting with 10 mg/kg/d up to 20 mg/kg/d in 8 days. After 1 week, he became more responsive (Figure 1E,F), and after 1 month, he was seizure‐free, and the EEG was significantly improved (Figure 1G,H). Epidyolex® was added to ethosuximide and clobazam which were not effective alone. After 4 months of follow‐up, no clinical‐EEG modifications were observed. The patients did not present adverse events both in the acute phase of administration and during the follow‐up.

This case has shown the potential benefits given by Epidyolex® CBD for the treatment of NCSE in a patient with AS. The faster titration was well tolerated.

Given the need for innovative treatments, especially for drug‐resistant epilepsies, Epidyolex® CBD may be a promising anti‐seizure medication and has been given “off label” to people with epilepsy syndromes outside LGS, DS, and TSC.^4^ Interestingly, acute CBD (100 mg/kg) treatment attenuated hyperthermia‐ and acoustically induced seizures in a mouse model of AS supporting the hypothesis that CBD may alleviate seizures and EEG abnormalities in AS, putting the basis for a rational development of CBD as treatment for epilepsy in AS.^5^ The use of CBD in refractory status epilepticus has been recently reviewed, and in 9 out of 11 treated patients the outcome was favorable.^6^

We believe this is the first report of the use of CBD in the acute treatment of NCSE in patients with AS. Although anecdotal, this observation ought to encourage further trials and confirmation from future studies.

## CONFLICT OF INTEREST STATEMENT

PN has received speaker fees or fundings or has participated in advisory boards for Angelini and UCB. DPL, AM, CT report no conflict of interest. VF has received speaker fees or fundings or has participated in advisory boards for Zogenix, Neuraxpharm, Angelini, Eisai, and Neuraxpharm. SN has served on scientific advisory boards for GW Pharma, BioMarin, Arvelle, Marinus, and Takeda; has received speaker honoraria from Eisai, Biomarin, Livanova, Sanofi; has served as an investigator for Zogenix, Marinus, Biomarin, UCB, Roche.
